# Cause and Mitigation of Lithium-Ion Battery Failure—A Review

**DOI:** 10.3390/ma14195676

**Published:** 2021-09-29

**Authors:** Muthukrishnan Kaliaperumal, Milindar S. Dharanendrakumar, Santosh Prasanna, Kaginele V. Abhishek, Ramesh Kumar Chidambaram, Stefan Adams, Karim Zaghib, M. V. Reddy

**Affiliations:** 1 Automotive Research Center, School of Mechanical Engineering, Vellore Institute of Technology, Vellore 632014, India; milindarsd15@gmail.com (M.S.D.); santoshprasanna@gmail.com (S.P.); kvabhishek065@gmail.com (K.V.A.); 2 Department of Materials Science and Engineering, National University of Singapore, Singapore 117575, Singapore; mseasn@nus.edu.sg; 3 Department of Mining and Materials Engineering, McGill University, Wong Building, 3610 University Street, Montreal, QC H3A OC5, Canada; karim.zaghib@mcgill.ca; 4 Hydro-Quebec Institute of Research (IREQ), Centre of Excellence in Transportation Electrification and Energy Storage (CETEES), Hydro-Québec, 1806, Lionel-Boulet Blvd., Varennes, QC J3X 1S1, Canada; 5 Nouveau Monde Graphite, 995 Rue Wellington, Suite 240, Monteral, QC H3C 1V3, Canada

**Keywords:** Lithium-ion battery, electrode materials, electrolyte, failure modes, failure mechanisms, mitigation

## Abstract

Lithium-ion batteries (LiBs) are seen as a viable option to meet the rising demand for energy storage. To meet this requirement, substantial research is being accomplished in battery materials as well as operational safety. LiBs are delicate and may fail if not handled properly. The failure modes and mechanisms for any system can be derived using different methodologies like failure mode effects analysis (FMEA) and failure mode methods effects analysis (FMMEA). FMMEA is used in this paper as it helps to identify the reliability of a system at the component level focusing on the physics causing the observed failures and should thus be superior to the more data-driven FMEA approach. Mitigation strategies in LiBs to overcome the failure modes can be categorized as intrinsic safety, additional protection devices, and fire inhibition and ventilation. Intrinsic safety involves modifications of materials in anode, cathode, and electrolyte. Additives added to the electrolyte enhance the properties assisting in the improvement of solid-electrolyte interphase and stability. Protection devices include vents, circuit breakers, fuses, current interrupt devices, and positive temperature coefficient devices. Battery thermal management is also a protection method to maintain the temperature below the threshold level, it includes air, liquid, and phase change material-based cooling. Fire identification at the preliminary stage and introducing fire suppressive additives is very critical. This review paper provides a brief overview of advancements in battery chemistries, relevant modes, methods, and mechanisms of potential failures, and finally the required mitigation strategies to overcome these failures.

## 1. Introduction

Internal combustion engines are a hundred-year-old technology and their development was backed by the stringent emission norms imposed by environmental pollution control boards in different countries. As the emission norms are becoming more stringent, internal combustion engines cannot meet the future norms. Hence, electric vehicles are the future automotive technologies with hybrid electric vehicles as a bridging technology. The electrification of the automotive sector is mainly driven by changes in climate-related policies, awareness of economic and security problems related to oil, a rise in pollution in the air which is depleting air quality, and rapid technology development in the field of batteries. Thus, there is a need to develop vehicles that run on sources other than fossil fuels. In electric vehicles, the internal combustion energy is replaced by a simpler and lighter electric motor that gets its power locally from the batteries, thereby, a wide variety of power sources including renewables can be used to supply the energy for the propulsion of the car. The electrification of the automotive sector, as well as ever-increasing demands for consumer electronics [1], have led to the substantial growth of Lithium-ion batteries (LiBs) [2]. In electric and hybrid electric vehicles, LiBs are extensively preferred over other types of batteries due to their tremendous performance and long shelf life [3,4]. Despite their advantages, LiBs have certain disadvantages that need to be examined. LiBs are sensitive to high power charging (fast charging), a too high or too low operating temperature, and mechanical abuse which eventually leads to capacity fade, short-circuiting, and the hazard of thermal runaway [3,5,6,7,8,9]. Repeated fast charging can expedite battery aging, resulting in shorter battery life. Xianke et al. investigated the effects of fast charging on the LiBs [10]. The optimal working temperature range for LiBs is 15 °C to 35 °C whereas temperatures above or below this range have a negative influence [11]. Low operating temperatures diminish battery capacity and power density [12], while high temperatures increase internal resistance and reduce active material availability, resulting in capacity and power loss [11]. Mechanical abuse and/or extremely high operating temperatures can result in short circuits that may lead to thermal runaway [11,13,14]. Based on the above-mentioned shortcomings, safety, and performance improvement studies of LiBs should go hand in hand and for this, it is necessary to understand the materials used in LiB construction.

A thorough understanding of each material will enable the researchers to improve the materials [9] according to the requirements and also helps to develop new materials to overcome the shortcomings of the existing battery chemistry. Studies to improve the existing battery materials and for the development of new materials are recent trends in the area of battery research. Due to increased demand, the development of safe and low-cost materials, along with improved performance, is needed at this time. To improve existing or developed new materials, it is essential to understand how the existing material can undergo failure. There are numerous ways by which a battery can fail. Analyzing those methodologies at the component level, as well as at the system level, will aid in the creation of safer batteries. A thorough understanding of the failure methods helps in devising strategies to mitigate the battery failures, thereby improving safety.

Mitigation strategies are critical to reducing the risk of failures in LiBs as well as their consequences. They can thus be achieved in two steps. In the first step, strategies are implemented during the normal operation of batteries, to reduce the risk of a particular failure mode occurring in a device. Such strategies include material alterations, protection devices, and thermal management of batteries. In the second step, implemented strategies are the ones that are adopted when the LiBs have failed to aid in evading the disaster subsequently caused by the failure. This includes the isolation, diagnosis, and extinguishing of fires.

**Figure 1:**
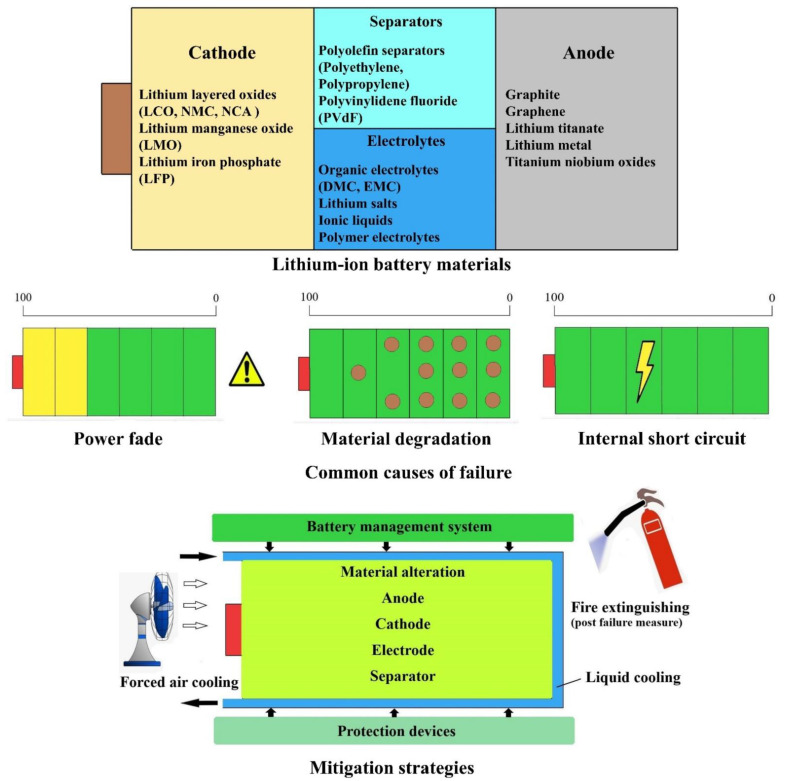
LiBs materials, causes of failure, and mitigation strategies.

The contents of this literature review have been organized into five sections. A schematic overview of the topics covered is also seen in Figure 1 below. After this introduction, Section 1 and Section 2 provide insights into the materials used for LiBs. It also provides information on material history, capacities, functions, merits, and demerits, and current studies. Section 3 gives an in-depth analysis of each component, discussing the mechanisms of failure of these components. These mechanisms explain why and how a component can fail, which might further lead to a system-wide breakdown. The mechanisms leading to failure for each component are explained using flowcharts. Furthermore, in Section 4, the possible modes of failure are discussed and are broadly classified into mechanical, electrical, and thermal modes. In each mode, all the possible scenarios and tests that validate the course of failure are discussed. Section 5 elaborates mitigation strategies that are classified into five modes; namely, (1) Innate Safety; (2) Protective Devices; (3) Thermal Management; (4) Mechanical Mitigation Strategy; and (5) Fire Control. The innate safety strategies are further classified into material alterations that are carried out in cathode, anode, electrolyte, and separators. Furthermore, in the innate safety section, battery management systems are discussed briefly. Subsequently, protective devices including protection vents, Positive Thermal Coefficient (POTC) devices, and circuit breakers are discussed before we provide an overview of temperature management strategies using air, liquid, and Phase Change Material (PCM) cooling. The mechanical mitigation strategies section then covers various types of battery casings to evade any form of failure due to mechanical loads. Finally, Fire Control mechanisms comprising the detection mechanisms and extinguishing agents are explained.

## 2. LiBs Materials

A rechargeable battery is an energy storage component that reversibly converts the stored chemical energy into electrical energy. LiBs are a class of rechargeable batteries that are capable of undergoing numerous charging and discharging cycles. They have gained rapid popularity in recent times due to their superior performance. Similar to other batteries, they comprise important components such as electrolytes (liquid, polymer, or solid) [15], separators [16], cathode [4], anode [17], current collectors, and casings. During the charging process, there is a movement of Li-ions from cathode to anode through the electrolyte, while the electrons flow through the external circuit in the same direction. During the process of discharge, the stored Li-ions move back from anode to cathode. In both processes, Li-ions are extracted from and stored in host materials and this process of ion insertion from one electrode to another is termed intercalation. Every component used will contribute towards battery performance and safety, and finding out the best possible combination will help in creating better batteries [4,18].

### 2.1. Anode

**Figure 2:**
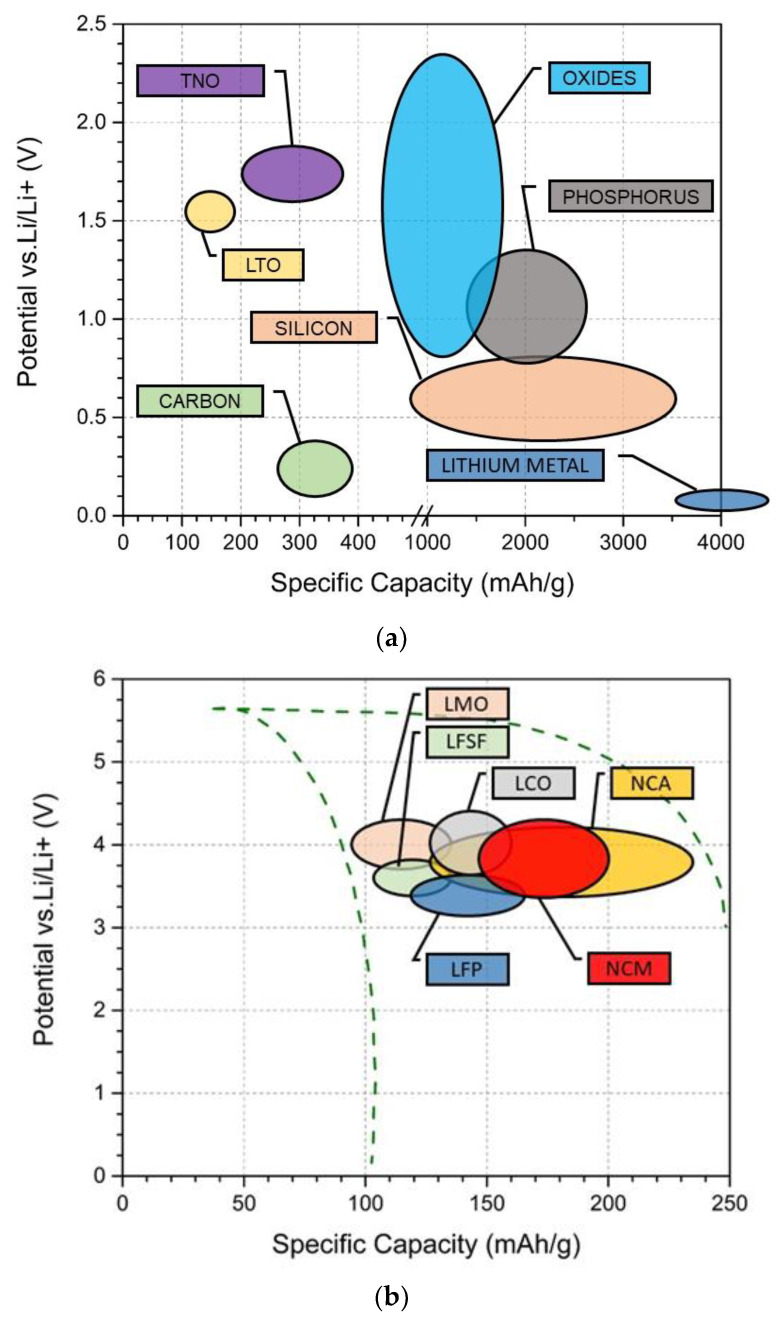
Discharge potential v/s specific capacity of some commonly used (**a**) anode and (**b**) cathode materials. Reproduced with permission from ref. [28]. Copyright 2014. Nitta, N., Wu, F., Lee, J.T., Yushin, G.; published by Elsevier Ltd.

The discharge potential versus capacity graph for the commonly used anode and cathode materials is shown in Figure 2. Anode materials should possess a lower potential, a higher reducing power, and a better mechanical strength to overcome any form of abuse [19,20]. Several materials such as graphite [4], carbon, and lithium titanate Li_4_Ti_5_O_12_ (LTO) [21] have been tried and tested for quite some time and a few, such as silicon, lithium metal, or titanium niobium oxides (TNO) [22], are still under research. However, there are certain drawbacks during normal battery operation, which would lead to the initiation of mechanisms that might cause anode material failure. These are discussed in Section 3.1. Graphite is widely used because of its layered structure, large insertion capacity (372 mAh∙g^−1^), high reversibility, and low cost. Moreover, the very low potential for Li insertion allows for a high cell voltage [23] but limits the fast-charging capability (due to the hazard of lithium metal plating). In graphene, the same monoatomic carbon layers are accessible individually, so that the theoretical specific capacity is almost thrice when compared to graphite, so that it has been considered as an anode material of the future, despite its higher price. Similarly, carbon nanotubes and fullerenes are under study for lithium storage [24,25,26]. LTO is another good anode material that has a defective Spinel structure and the research on LTO has gained prominence over the years [27]. The thermal stability of LTO is considerable [28], and the material does not exhibit any volume change during intercalation/deintercalation [29] so that it is also referred to as a Zero Strain (ZS) material [19]. The spinel structure provides a three-dimensional network of Li+ ion pathways with low migration barriers that further promote the rate capability of the LTO anode. Its high potential (1.55 V) enhances safety on fast charging, but along with the low specific capacity of about 150 mAh∙g^−1^, limits the achievable energy density [30]. A detailed literature review on LTO chemistry was carried out by Zhao et al. and can be referred to, to obtain additional information [27]. More recently, a series of Titanium Niobium Oxides Ti_x_Nb2_y_O2_x_+5_y_ (e.g., TiNb_2_O_7_, TiNb_6_O_17_, TiNb_24_O_62_) have been explored as an alternative to LTO for high-rate applications, as they combine the elimination of the Lithium plating hazard due to a high Li insertion potential (ca. 1.6 V) with a higher theoretical capacity that can be practically accessed even at high rates by carbon coating and doping. For details, the reader is referred to a recent review by Yuan et al. on this class of material [22].

Lithium alloying elements [31], such as Silicon (Si), Tin (Sn), Antimony (Sb), Zinc (Zn), Phosphorus (P), Germanium (Ge), Bismuth (Bi), and Aluminum (Al), are also considered as potential anode materials. Silicon material has a very high specific capacity and a lower voltage potential compared to other materials, which is the essential requirement for achieving high energy density [20,23]. However, during the lithiation and delithation process, materials like silicon and tin undergo huge volume expansions around 300%. This damages the electrode microstructure accelerates capacity fading and may lead to short-circuiting and as a consequence thermal runaway [32]. Therefore, to avoid hazards, silicon is converted to either an alloy or a composite where nanostructured silicon or its alloys are combined with graphite, graphene, or other forms of carbon, silicon and carbon nanotube composites, or silicon nanowires [23]. Zuo et al. [33] have provided in-depth information regarding the potential of silicon as an anode material.

Lithium metal itself has the highest specific capacity (>3860 mAh∙g^−1^) and the lowest potential (−3.040 V), which should make it an ideal anode material [34]. However, lithium is a highly reactive metal and formally the relative volume expansion trivially becomes infinitely large in this case. Since lithium is reactive in nature, the selection of suitable electrolytes is critical. Due to the large anode volume changes, the Solid Electrolyte Interface (SEI) layer can crack and dendrites formed during lithium cycling can grow through this layer, leading to short circuit and battery failure. Detailed reviews have been conducted by various authors on the potential of lithium metal as an anode material [34,35,36].

### 2.2. Cathode

Generally, LiBs are identified by the cathode material chemistry as the choice of the material used as the cathode will mostly dictate both the battery performance and its cost. Early attempts for intercalation concepts were made in 1970 [16,37]. Since its inception in the 1980s on layered cathode LiCoO_2_, several chemistries have been tried and tested. Popular chemistries and widely preferred. Among them is lithium cobalt oxide LiCoO_2_ (LCO), lithium nickel manganese cobalt LiNi_x_Mn_y_Co_z_O_2_ (NMC), lithium manganese oxide (LMO), lithium nickel cobalt aluminum (NCA), and lithium iron phosphate, LiFePO_4_ (LFP).

LCO was introduced in the year 1980 by Mizushima et al. [38] and was the first chemistry to be commercialized [3]. It is a type of layered transition metal oxide that possesses hexagonally shaped crystal platelets [39]. Assuming full utilization of the lithium contained, it has an attractive theoretical specific capacity of almost 274 mAh∙g^−1^ [29], but practically, the capacity is limited to about 155 mAh∙g^−1^ [40] due to its limited structural stability [41]. The average operating voltage is 3.9 V [42] as further delithiation would destabilize the structure. Its advantages include a high working voltage, high mobility of electronic and ionic charge carriers, a good cycle performance, and a high energy density [43]. Its few drawbacks include its high cost and the supply limitations of cobalt [40], and moderate thermal stability [44]. The introduction of Nickel and Manganese into LCO created Li(Ni_x_Mn_y_Co_z_)O_2_ (x = y = z = 1) (NMC) cathode materials [45,46]. This enhances practical capacity and mitigates reliance on supply-critical cobalt. Alternatively, the Nickel-rich compositions can be stabilized by electrochemically inert aluminum, leading to LiNi_0.8_Co_0.15_Al_0.05_O_2_ (NCA) with an enhanced capacity and cycle life [47], while the introduction of metal oxide coatings improved cyclability [48]. However, the cost, toxicity, and supply limitations of cobalt demand newer and better materials to be developed to create sustainable batteries.

LiMn_2_O_4_ (LMO) was introduced in 1983 by MM Thackeray et al. as one of the potential cathode materials. It has a Spinel structure [49]. With lower theoretical (150 mAh∙g^−1^) [50] and practical (<20 mAh∙g^−1^) capacities [51], it is comparatively inferior to LCO. However, the average operating voltage (4 V) is similar to LCO. This particular chemistry is stable with lithium extraction [52] and due to the abundance of manganese is more economical than LCO [50]. On the other hand, capacity fade at high temperatures [53] and oxygen loss [54] are key factors limiting the current application range of this chemistry to low-cost LIBs. Partial replacement of manganese with aluminum, however, improves cycle performance [55,56], nano-composite techniques improve specific capacity [57] and surface modification techniques improve the capacity retention at a broader temperature range [58]. It is expected that manganese-based cathode chemistries will gain importance simply because the supply limitations of alternative transition metals (cobalt and increasingly also nickel) become more prominent with the scale-up of LIB production.

LFP is a relatively newer cathode material that was introduced by A. K. Padhi et al. in the year 1997 [59]. It possesses an olivine-type framework structure, leaving one-dimensional channels for Lithium ions. Its practical capacity (<165 mAh∙g^−1^) is close to the theoretical value (170 mAh∙g^−1^) [60]. It is environmentally safer when compared to others with good cyclability [61] and high thermal stability due to its strong P–O bond [28], making it potentially a very good cathode material. The use of highly abundant iron as the redox-active transition metal makes it also a highly sustainable cathode material. However, it possesses a low voltage (3.4 V) [60], ionic, and electrical conductivity [62] in comparison to the materials discussed above. Multiple types of research are being carried in the field of LFP chemistry to improve the existing drawbacks. The reduction in particle size helps to achieve better chemical performance [63]. Doping of materials like nickel, cobalt, and magnesium improves battery performance and cyclability [64]. Moreover, other materials, such as triclinic structure (LiVPO_4_F) [65], Vanadium-based metal inorganic or organic phosphate (PO_4_), phosphite (HPO_3_), and oxalates as cathodes [66,67,68,69], and other tavorite structures called fluorosulphates, are being currently developed which could potentially replace LFP [70,71]. As mentioned in the above paragraphs, each of the cathode materials come with their own set of drawbacks, and these can initiate certain processes that will eventually lead to the cathode failure mechanisms that are discussed in detail in Section 3.2.

### 2.3. Electrolytes

There has been a rapid increase in the working voltage potential of the LiBs that has forced researchers to explore safer and better ion-conducting materials that can be used as an electrolyte [15]. During the charging and discharging process, ions move from one electrode to another and the electrolyte acts as a conducting medium. There are certain criteria that materials should meet to be used as an electrolyte. (1) it should allow only specific ions but not the electrons to pass; (2) it should not undergo degradation over a wide range of working potentials; (3) it should not react with other battery components; (4) it should be thermally stable over a wider range of operating temperatures; (5) it should be non-toxic, sustainable, environmentally friendly as well as economical [72]. Along with battery reactions that cater to the charging and discharging process, certain side reactions lead to a slump in battery capacity and increased wear. This phenomenon is discussed in detail in Section 3.3 while this section is focused on the materials that are widely used as electrolytes currently.

Salts of lithium dissolved in electrolytes based on organic carbonate compounds have been widely used since LiBs were first commercialized. Popular solvents are mixtures of organic carbonates and esters such as Propylene Carbonate (PC), Dimethyl Carbonate (DMC), Diethyl carbonate (DEC), and Ethylmethyl Carbonate (EMC). To enhance ionic conductivity, salts with weakly coordinating anions were added. The most commonly employed Lithium salt in commercial LIBs is Lithium Hexafluorophosphate (LiPF_6_). Most alternative salt anions such as ${\mathrm{BF}}_{4}^{-}$, trimethanesulfonate (Triflate), bis(trimethanesulfonyl)imide (TFSI), etc., also contain fluorides to enhance the electrochemical stability. As the processing of fluorinated compounds comes with hazards, various fluoride-free materials have been explored (e.g., bis(oxalate)borate (BOB), tetracyanobaroate (Bison), etc.) but none of them could achieve the cost-performance ratio of the fluorinated competitor LiPF_6_ when applied to graphite-based LIBs. However, for next-generation batteries with alternative anodes, different Li-salts may be more advantageous [73]. Fluorinated organic carbonates enhance the cycling stabilities compared to non-fluorinated carbonate electrolytes [74,75]. Kang Xu. [76] carried out extended research on lithium salts and organic electrolytes. Even though organic solvent mixtures are widely used in commercial batteries, the volatility and flammability characteristics of organic solvents cause concern over the safety of the battery. The focus now is towards the usage of non-flammable electrolytes like ionic liquids and polymer electrolytes [23]. Ionic liquids have better stability, a larger operating temperature range, and higher ionic conductivity compared to organic electrolytes [72]. Imidazolium, quaternary ammonium, pyrrolidinium, and piperidinium-based ionic liquids are some of the commonly used ionic electrolytes. Prominent research [77,78,79,80] is being carried out in the areas of synthesis and performance enhancements of ionic electrolytes. The newer electrolytes, comprising polymers and polymer electrolytes (PoE), can be broadly classified into solid and gel. Gel PoEs will have lithium salts and solvents confined in a polymer matrix (physical gel PoE) or with a chemical bond and cross-linking agent (chemical gel PoE). Solid polymer electrolytes are obtained by dissolving salts of lithium in a polymer matrix with an electron donor group. Polymer electrolytes are flexible, safe, and have a higher ionic conductivity and mechanical strength [81]. More information in detail related to PoEs can be found in [81,82].

### 2.4. Separators

A separator is a thin porous membrane that separates two battery electrodes and stores a certain amount of electrolyte to aid in ionic transport while preventing short-circuits by direct contact with the active materials [83]. The early history of separators is discussed in a recent review [16]. Separators should not undergo any electrochemical reaction, but their presence is key in battery safety and performance. Several factors are to be taken into account to select a suitable separator [29]. Important characteristics of separators [16,84] are (i) good electrochemical stability; (ii) strong electrolyte absorption and retention capabilities (i.e., high wettability by the electrolyte), which improves ion transport and hence cell performance; (iii) optimum thickness 25 μm, low thickness yet high puncture strength to survive the significant mechanical stresses during battery fabrication and operation. Moreover, they lower the distance between anodes and cathodes, and thereby the internal resistance, improving both volumetric and gravimetric energy density, (iv) a significant amount of porosity (40%), an appropriate pore size, and higher permeability. Porosity is necessary to trap the electrolyte, but a higher porosity could adversely affect the separator performance and the pore distribution should be as uniform as possible [29]. Pore size should be small enough to block electrode materials or dendrites. Permeability is measured in terms of the MacMullin number, which gives the ratio between the resistance of the separator soaked with the electrolyte and the free electrolyte of the same geometry [85]. The lower the MacMullin number, the better the separator’s performance [86], but high mechanical strength and an adequate porosity at around 40% are the most important properties that determine how good the separator membrane is [68] ((v) good thermal and dimensional stability). The separator should not curl up or shrink when the temperature within the cell increases during operation. For further information on performance evaluation, literature work [87] can be referred to. Several conventional and modern methods are used to manufacture separators. Wet and dry methods are widely used in large-scale manufacturing and a detailed review of manufacturing methods can be found in [86]. The electrospinning technique is widely used to develop newer nanofiber-based separators. Li et al. [83] have carried out a comprehensive review of electro-spun nano-fiber separators, their advantages, necessary improvements, and materials. Membranes can be broadly classified into the micro-porous, non-woven mat, composite mat, and electrolyte types. For detailed information on the same, literature work by Lee et al. [85] and Francis et al. [88] can be referred to.

Some electro-spun separators are polyethylene terephthalate (PET), polyacrylonitrile (PAN), polyvinylidene fluoride (PVdF), and polybutylene terephthalate (PBT). These polyester-based separators will have a higher porosity when compared to Polyethylene (PE) and Polypropylene (PP), which are widely used currently in LiBs. PE and PP-based separators will have a thickness in the range of 20–25 µm, a porosity ranging from 36 to 48 percent, and the manufacturing of these separators can be either a wet or dry process. PE separators start to soften at 110 °C and melt at approximately 135 °C, while PP-based separators soften at 140 °C and melt at 165 °C. Advancements in this field include tri-layer separators, ceramic-coated separators, and polyester-based (non-woven) separators. Tri-layer separators are also called shutdown-type separators, which will have a PP layer sandwiched in between two PE layers, and are manufactured using a dry-type process. It possesses a similar thickness to that of single-layer separators. Ceramic-coated separators will have a polyolefin layer and an additional alumina layer coating on either side of the layer. Due to this coating, the ceramic-coated separators can withstand higher temperatures (above 200 °C) before melting sets in [86,89,90,91,92]. The temperature at which a separator should soften and thereby typically close its pores to become non-conducting should ideally be adapted to the temperature at which the thermal runaway sets in so that the separator can help to prevent fire hazards.

The advantages of the above-mentioned separators are explained in Section 5.1.4. However, they are sourced from crude oil. Coping with the development and usage of sustainable materials in batteries, the potential of several biopolymer fibers such as hemp, flax, coconut, etc., are currently being explored and detailed information on the same can be found in [93]. Failures in separators happen mainly due to external factors or abusive usage. This phenomenon is explained in detail in Section 3.4.

## 3. Mechanism of Failure in LIB

**Figure 3:**
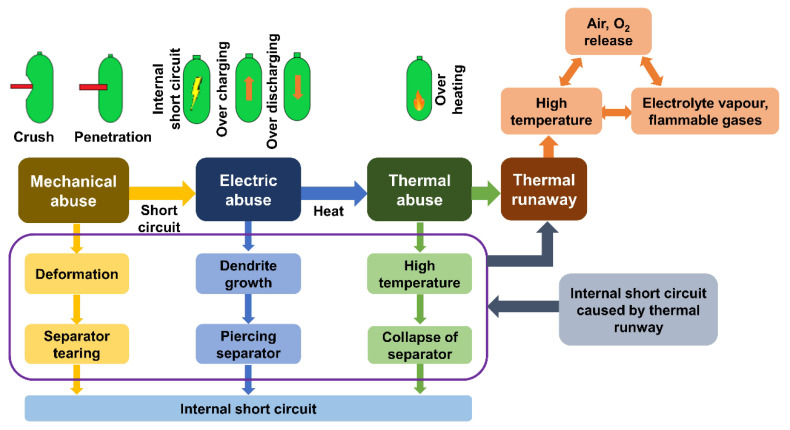
Scenarios that lead to Lithium-ion battery failure. Reproduced with permission from ref. [99]. Copyright 2019. Elsevier Ltd.

To study the modes of failure in LiBs, it is essential to study the process, cause, outcome, and mechanism of a particular failure that will affect the system’s performance. Failure Mode Methods Effects Analysis (FMMEA) is a symmetric method that involves identifying all probable modes of failure and their mechanisms. However, the mainstay feature of this method is its ability to even involve the Physics of Failure (POF) point of view to assess the reliability at each level, be it system, sub-system, or component level. This gives an advantage over Failure Mode Effects Analysis (FMEA), where only methods are considered and not the reasons or the path that led to the particular failure [94]. The usual mechanisms that lead to failure are physical, chemical, electrical, and mechanical in nature [95]. Hendricks et al. [96] reported that the above mechanisms help identify the path or the way a particular system fails due to one of the possible mechanisms. Failure modes—the particular ways in which a system can fail—can only be defined clearly when they are observed in real-time. A detailed study of the failure will reveal what the origin of the failure is. This cause of origin can be a product of intrinsic or extrinsic stresses that affect the system. Figure 3 below shows the possible scenarios that lead to incidents compromising the safety of the operation of LiBs. The method FMMEA can be implemented to the individual LiB cells and commercially available battery packs. This method requires years of experience acquired from multiple battery dismantling, reliability tests, and failure studies [97]. Using this method, component-level testing and assessment can be accomplished and it provides exhaustive details on the reasons regarding how each component can fail and contribute to malfunction or failure of the system.

### 3.1. The Active Material in Anode

For this review, as discussed in Section 2.1, only carbon-based anode materials were considered as they are most widely used [96]. A LIB eventually loses capacity, partially because of the reactions between the organic solvents like Ethylene Carbonate (EC) or DMC and the graphite [98]. The solvent also reacts with the anode, forming solid or liquid coagulation called a Solid Electrolyte Layer (SEI). This results in an irreversible loss in capacity, as well as in achievable power. This SEI layer is permeable and allows the movement of ions, so it will grow over time, which further deteriorates the battery and its life span. This layer formation is also spread to other parts of the cell [96]. The mechanism of failure in the case of anode material failure is due to the reduction reaction at electrodes leading to power fade in batteries. Upon observation, as the reaction proceeds, the thickness of the SEI layer is increased.

This SEI layer is highly delicate and it is very challenging to dismantle the battery without causing damage to it. Therefore, partial disassembly is done and the mechanism is studied using Nuclear Magnetic Resonance (NMR) imaging [100]. If the analysis needs to be carried out, then it requires highly tedious multiple processes to study the behavior of the SEI layer [101]. Formation of this layer is not the key factor leading to the failure, but it is the decomposition of this layer at increased temperatures that affects the health of the battery, making way to the thermal runaways that follow after the release of gases [102]. Here, the generation and growth of the SEI layers are seen as failure due to wear, which results in reduced battery capacity and increased internal resistance according to FMMEA.

Dendrite growth is another issue related to the anode–electrolyte interface and is a highly critical mechanism of failure [18]. Dendrites puncture the separators and lead to Internal Short Circuits (ISCs). Dendrite growth usually occurs due to quick or rapid charging of the battery at a sub-operating temperature range (less than 20 °C) [103]. NMR can be used to efficiently detect the dendrite growth in the Li-ion cells [104]. This operando method is preferred over cell dismantling mainly due to the instability of the dendrites. During the process of dismantling, if any short circuit occurs, all the dendrites will disappear, leaving behind little trace of formation or existence [105].

**Figure 4:**
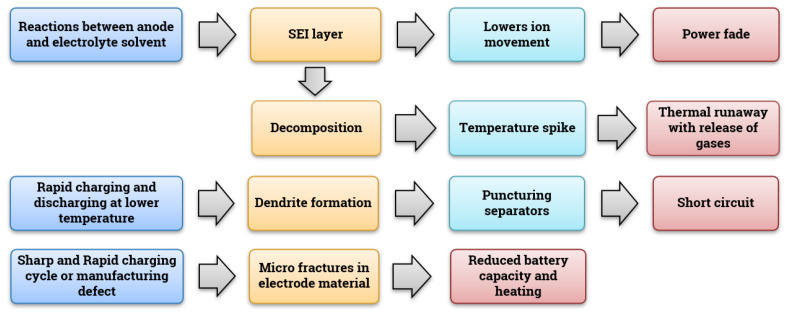
Anode active material failure mechanism.

Apart from these key failure mechanisms, one more failure that hampers the working of the battery is the fractures observed in particles of the electrode, due to the formation of micro-sized cracks on the surface of the electrode material. This problem arises in the case of a sharp and rapid charging cycle, poorly designed and sized electrode particles, and their uneven distribution [106]. Volumetric expansion or contraction is also a reason for instability in various parts of the electrode. To overcome these failures in the anode, certain strategies are adopted, and these are mentioned in Section 5.1.1. The Li and graphite particles are the ones that combine in a reaction that is intercalating in nature. All these observations are done using the Focused Ion Beam (FIB) technique, Scanning Electron Microscope (SEM), and X-ray nano tomography [107,108]. Figure 4 depicts the various important possibilities for anode material failure.

### 3.2. Cathode Material

**Figure 5:**
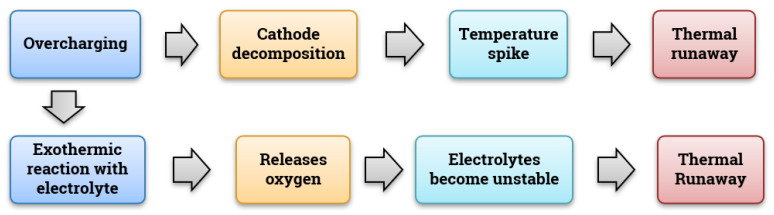
The possible failure mechanism of the cathode.

Usually, the cathode is made of lithium oxides of cobalt (LiCoO_2_), LiNi_x_Mn_y_Co_z_O_2_ (NMC-811,622), manganese (LiMn_2_O_4_), LiMn_1.5_Ni_0.5_O_4_, or iron phosphate (LiFePO_4_) [59,109]. Cathodes are also susceptible to the SEI layer but are not to the degree when compared to the reaction with graphite at voltages higher than 4.5 V (because the film growth is stable at higher voltages) [110] and even decomposition is observed in the case of over-charging. Under the over-charge condition, the electrolyte undergoes an exothermic reaction with the cathode material generating oxygen, making the electrolyte unstable [111]. The origins and mechanisms of gas generation are still under discussion. Mitigation strategies to avoid the above-mentioned failures are discussed in Section 5.1.2. Figure 5 denotes the failure mechanism for the cathode material.

### 3.3. Electrolyte

**Figure 6:**
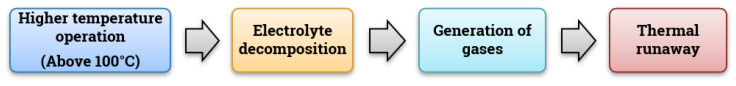
Electrolyte failure mechanism.

Organic-based solvents with dissolved salts containing lithium are used as electrolytes in LiBs. One of the most commonly and commercially used electrolytes is EC and DMC, which are organic carbonates. They are one of the main contributors to the side reaction taking place at the electrodes, leading to a slump in the battery capacity and wearing. Though the widely used electrolytes are highly efficient in ion transmission, they possess the drawbacks of being highly inflammable and unstable, except for a limited window of temperatures and voltages. Any operation under extreme conditions (typically above 100 °C) will result in decomposition and the generation of gases leading to thermal runaways [112]. The rate and level of degradation of the electrolyte in use can be compared with that of an unused electrolyte can be studied using processes like Differential Scanning Calorimetry (DSC) [113], Accelerated Rate Calorimetry (ARC) [114], NMR [115], Fourier Transform Infrared Spectroscopy (FTIR) and Thermo-Gravimetric Analysis (TGA) [116]. Figure 6 shows the failure path for electrolytes. As mentioned above, the flammability issue can be curbed using certain strategies which are discussed in Section 5.1.3.

### 3.4. Separators

**Figure 7:**
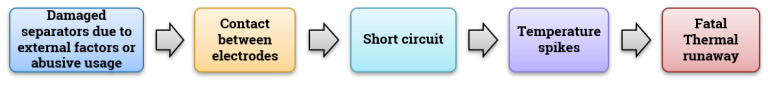
Separator failure mechanism.

Separators in LiBs are made of porous electrolyte-soaked polymer sheets to facilitate ion transmission between the electrodes while preventing their direct contact. If these separators are damaged, the gaps between electrodes cease to exist, leading to short circuits and temperature spikes, finally resulting in a fully-fledged thermal runaway [84]. Damaging of the separator can be due to the growth of Li-dendrites or external factors like mechanical abuse during cell assembly or operation. Any damage to separators can be studied either by using SEM or bare eye investigation of melted separators or can also be done by disassembling the cell. Studies are being carried out to study the origin of stresses in the separator caused over time during normal operation or any form of abuse [117,118]. Abuse leads to the melting of the polymers used in separators, causing a very dangerous thermal runaway scenario [119]. Battery aging is also one of the contributing factors for separator failure. A study conducted by Zhang et al. [120] shows that several factors influence aging in battery separators, namely, hysteresis in temperature and mechanical loading, oxidation of separator film, accumulation of chemical particles in pores of the separator film, and the effect of electrolytes. Figure 7 depicts the stages leading to separator failure.

Orendorff et al. [89], in their study, pointed out that temperature variance between the cells in the larger battery packs is very high when compared to that of smaller battery packs. This uneven temperature profile will cause heterogeneous degradation of separators, which in turn causes uneven cell resistances within the battery pack. More research must be carried out in this area. However, certain measures are introduced by various researchers to mitigate the failure in the case of separators and one of them is the shutdown type of separator. The details about shutdown type separators and prevention of dendrite growth in anodes by altering the material of separators are described in Section 5.1.4.

### 3.5. Current Collectors

The anode is usually coated with etched copper foil that acts as a current collector for an anode and it is inert to the chemical reaction. Whereas if we use Cu-foil as a cathode current collector when we cycle above 3.5 V, oxidation occurs, then it may lead to degradation in battery performance [121] and meltdown due to the over-discharging scenario [96]. Over-discharge is a phenomenon that occurs when a cell is discharged beyond the safe voltage limit. Over discharging induces serious problems in larger battery packs [122]. The main cause for this type of failure is improper energy management in batteries or failed Battery Management Systems (BMS) or abusive usage of batteries [123].

On the cathode side, aluminum is used as a current collector. Again, the aluminum can undergo corrosion in the case of overcharging. Overcharging is a common failure, and it occurs when the charge current is forced through the battery even after it has reached a normal cut-off voltage [124,125]. Aluminum corrosion can lead to (i) an increase in electrical resistance; (ii) an increase in self-discharge rate due to the decomposition of the electrolyte [124]. Small contamination, such as ion metal impurities inside the cell, cannot be safely avoided during the manufacturing of batteries. Such impurities may cause short circuit defects and in the long run, lead to thermal runaway during overcharging. In batteries, overcharging dominates in the form of critical performance characteristics, and generally, there is no capacity degradation up to 120% overcharging in NMC and LMO cells. With NMC and LMO, a slight overcharge will thus not lead to a thermal runaway and not even to performance degradation. In contrast, overcharging of 105 to 120% already leads to surface temperature increases and capacity loss in LFP. Generally, overcharging is considered the most severe safety concern, and it is usually caused by the malfunction of the charging control system, inappropriate design of BMS, and even the capacity inconsistency in the cells. Hence, developing the methods of efficiently controlling voltage plays a crucial role in resolving the safety issues of LiBs.

**Figure 8:**
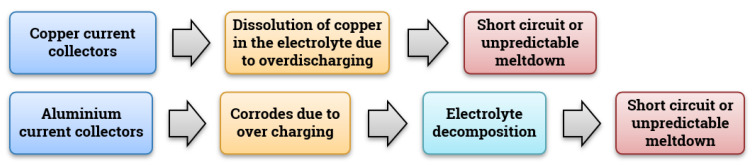
Current collector’s failure mechanism.

Hence, both overcharging and over-discharging of a battery must be avoided as much as possible. Figure 8 shows how the fault leads to failure for current collectors.

### 3.6. Cell Tabs and Casing

The current conduction to the external circuit is taken care of by the tabs of the cell. They are joined with the collectors using spot welding. Any mistake in the welding (like the lesser number of welded spots) will lead to a rise in contact resistance and lead to an increase in resistance to the flow of the current. Thermo-mechanical fatigue will result in connection failure between the tabs and cause the current connector to break down. Loose connections of any kind will result in total system malfunction or system-wide failure. Dampness in the casing where the battery is placed can result in short-circuiting. It can also lead to corrosion of the battery tabs, resulting in ponderous discharge and heat generation [126,127,128].

## 4. Failure Modes of LiBs

### 4.1. Mechanical Mode

So far, we have discussed the failure mechanisms involved and how each component of a battery has its role in the failure. These mechanisms may lead to or may be the cause of, certain modes of failure. The mechanical mode of failure appears to be the most perilous one, compromising the battery safety in case of a mishap [129]. In this mode, the battery or the casing undergoes deformation due to external loads that are mostly impulsive in nature. The load from the casing gets transferred to internal components of the battery, rendering them deformed. This leads to failure of the internal components such as separators and contact is attained between separator and cathode, which was studied by J. Zhu et al. [130] both by experimentations and simulations for 18,650 cylindrical cells. Numerous tests have been conducted to realize the behavior of both cylindrical and prismatic batteries and the conditions under which they undergo abuse. They can be differentiated as either collision/crush or penetration. Experimental methods for mechanical abuse of cylindrical and prismatic battery types are briefly summarized below.

#### 4.1.1. Cylindrical Battery

Single cylindrical cell or multiple cell batteries are subjected to multiple tests like the axial/radial compression/indentation, single point and triple or three-point bending, surface indentation, piercing, and Pellini test (commonly called as drop weight test). In a research project carried out by J. Xu et al. [131] for understanding the load-bearing capacity of cylindrical batteries in the radial direction, it was found that the ultimate loads that 18,650 batteries could bear were 40 kN and 602,030 cells could bear a load of 400 kN. Comparatively, indentation generates more local deformation in the radial direction despite the magnitude of the ultimate load being lower than the others (deformation). In this case, the dimensions of the indenter play a major role [8]. The deformations in the structures for three/triple point loadings were complex in nature. Here, the top layer underwent compression, but the bottom layer underwent tensional deformation. The max load that 18,650 cells could bear was just 4 kN and 602,030 cells could resist 100 kN [8,131].

#### 4.1.2. Prismatic Battery

These types of batteries are made to undergo slightly different tests due to their shape. They are subjected to various tests, such as compression or indentation, that were carried out considering deformation and stresses in just the z-direction or just the x-y direction and neglecting the other [132,133]. Bending and Pellini tests were also conducted but are not effective to lead ISCs as compared to other tests, due to the ability of the structure to bear high magnitude bending deformations. Dynamic loading tests, like the Pellini test [133] and impact tests for high velocity, Ref. [134] help study the structural behavior under dynamic conditions.

**Figure 9:**
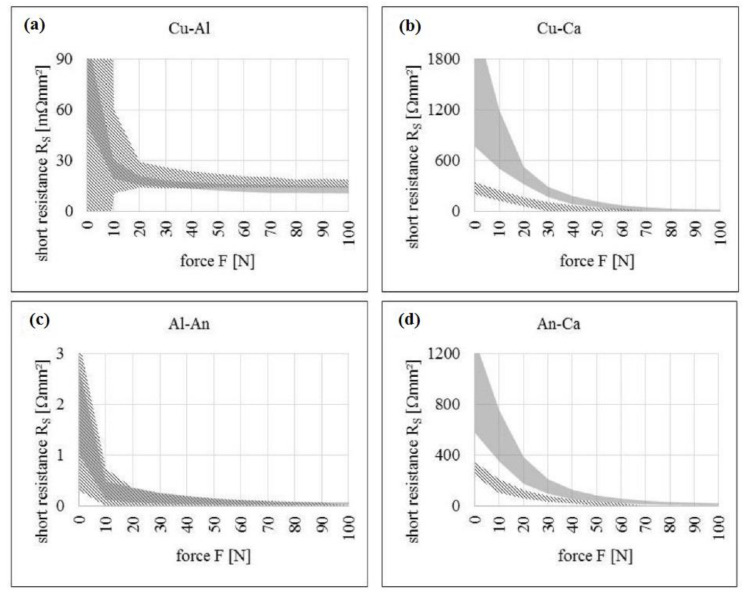
Plots of areal resistance (*y*-axis) vs. force (*x*-axis); (**a**) Cu–Ca contact scenario, (**b**) An–Ca contact scenario, (**c**) Cu–Al contact scenario, (**d**) Al–An contact scenario. Reprinted from ref. [135].

All these tests help envisage the functions of the battery under those scenarios and understand how it will further deteriorate the battery condition. In all these tests, the outcome was ISCs. This was mainly due to the formation of contact between the internal components of the battery. The mechanical stresses generated during this mode decide the reactions of ISCs. This fact has been witnessed multiple times in several studies such as e.g., LFP. In the case of Cu–Ca, Figure 9a shows that for higher forces, the combination shows good accordance with >>100 m ohm. At higher loads, the particles penetrate the electrolyte layers and establish direct contact with the current collector. Figure 9b depicts the An–Ca contact scenario. The values of short resistances show good accordance with >>100 m ohms for higher forces. The results of a Cu–Al contact scenario are as shown in Figure 9c. It is observed that with an increase in the force, the short resistance value decreases to around 10 m ohm and maintains it for higher force values. For Al–An, Figure 9d shows that for force values above 20 N, the particles exhibit good accordance with the ~100 m ohm. Finally, we can summarize that electrolyte can influence the resistance of Lithium Ferrous Phosphate [135,136,137]. The mechanical mode of failure is thus regarded as the initiator or propagator of the electric and thermal modes that are discussed in the following sections. Different battery casing structures, namely Navy Truss (NavTruss) and Blast Resistant Adaptive Sandwich (BRAS), help in mitigating mechanical failures like compression and penetration, which are discussed in Section 5.4.

#### 4.1.3. Pouch Cells

**Figure 10:**
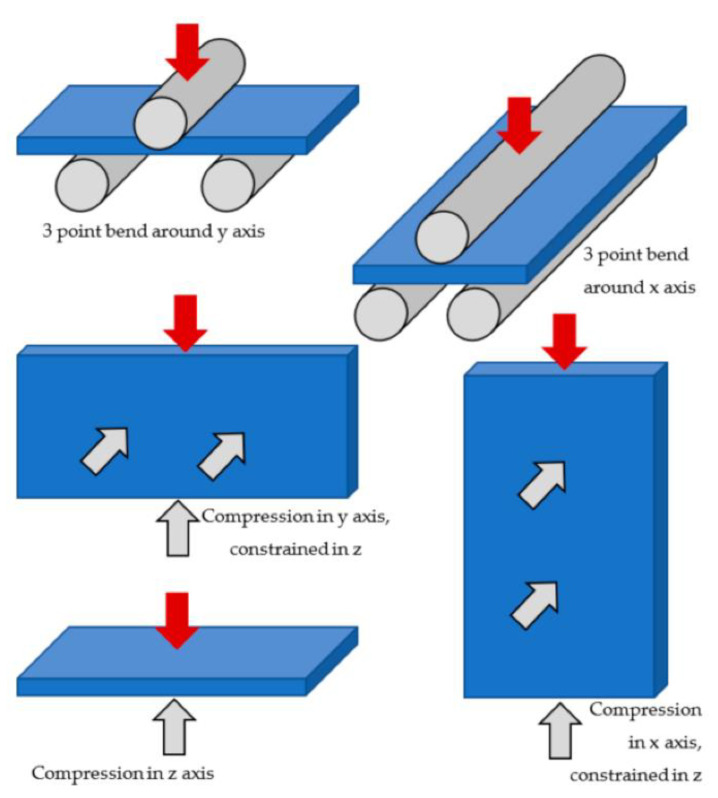
Load cases for physical testing of pouch cells. Reprinted from ref. [138].

**Figure 11:**
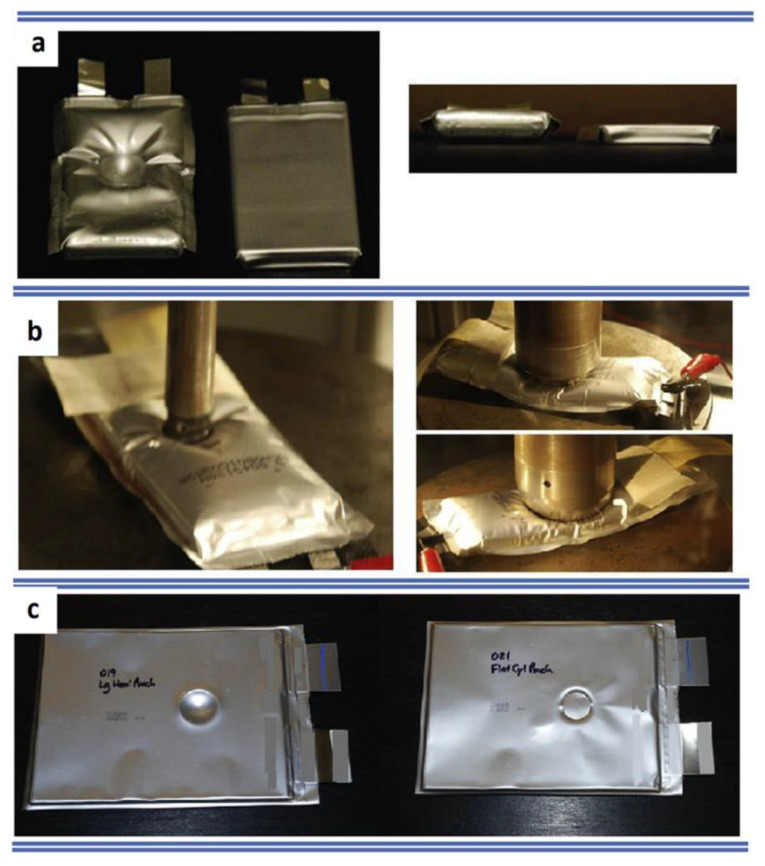
(**a**) Inflation of small pouch cell due to hemispherical punch indentation, (**b**) Inflation of medium pouch cell due to hemispherical punch indentation, (**c**) Inflation of large pouch cell due to hemispherical and cylindrical punch indentations. Reproduced with permission from ref. [139]. Copyright 2013. Elsevier B.V.

They are similar to prismatic batteries but without any solid enclosure. Pouch cells are often called soft packs. Pouch cells were tested using the Pelini test and compression test by Beaumont et al. [138] as indicated in Figure 10. Indentation tests are more effective and it was observed that the small (59.5 mm × 34 mm × 5.35 mm) and medium (129.5 mm × 43.5 mm × 8.2 mm) sized pouch cells are easily susceptible for ISCs compared to large ones (227 mm × 160 mm × 7.25 mm). Sahraei et al. [139] conducted tests for different-sized punches (Figure 11). It was observed that conical punch indentation tests induced ISCs more quickly compared to others, whereas the hemispherical punch with a smaller diameter could induce ISC more rapidly than a larger one. Therefore, it could be inferred that batteries can handle the intrusion of blunt objects well.

### 4.2. Electric Mode

The electric mode of failure can be observed as an independent event or the outcome of a mechanical mode failure. Again, ISCs are the common outcome of this mode of failure. The major electric signature of ISCs is the rapid drop in battery voltage due to contact between the internal active components of a battery [8]. Santhanagopalan et al. [140] in their work mention four possibilities of contact modes that result in ISCs. Table 1 below gives the details regarding these contacts and the severity of damage they can inflict. Earlier mentioned factors include the mechanical stresses leading to contact formation between different components. This further assists in the melting of the component materials which complicates the ISC mechanism. All the factors mentioned lead to this scenario of ISCs and they are also strongly influenced by mechanical behavior, as the stresses generated depend on the type of loading and its direction. To prevent a surge in current flow due to abnormalities in the cell assembly, as summarized in Table 1, protection devices inhibit the excess current flow, as discussed in Section 5.2.

### 4.3. Thermal Mode

**Figure 12:**
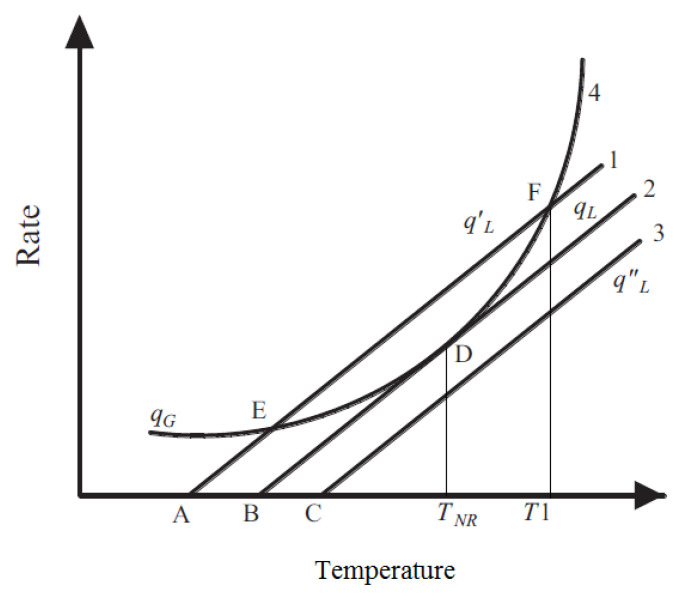
Semenov plot depicting heat generation and loss from a system. Reproduced with permission from ref. [142]. Copyright 2012. Elsevier B.V.

The thermal mode of failure normally occurs due to other modes that initiate this mode. LiBs are very susceptible to temperature. They are best operated in a narrow range of 20−40 °C (±5 °C). If this temperature interval is exceeded, then not only their performance is lowered, but their state of health also deteriorates. This is observed even in the case of a high atmospheric temperature. Prolonged usage at higher temperatures can lead to thermal runaway, further leading to the release of gases or an explosion and fire. As soon as the local temperature crosses the critical value, this initiates multiple side reactions (discussed in Section 4.3.1) that are exothermic in nature, increasing the temperature further. If uncontrolled, this temperature increase will lead to thermal runaway [13]. The dynamics of these side reactions leading to thermal runaway can be studied using the Semenov plots [141] as shown in Figure 12. As soon as the local temperature crosses the critical value called “Temperature of no return” (TNR) [142], an uncontrollable avalanche of exothermic side reactions (discussed in Section 4.3.1) sets in [143].

Irrespective of working and boundary conditions, the LiBs will eventually reach the value of TNR on uncontrolled thermal abuse, from which point onwards a thermal runaway becomes unavoidable. This method only simplifies the study with the assumption of uniform temperature distribution [99]. The governing equations discussed in Section 4.3.1 for each component show how each of them behaves under thermal runaway conditions.

#### 4.3.1. Governing Equations for Thermal Runaway

##### Anode

The metastable components of SEI undergo decomposition between 80 and 120 °C and a maximum decomposition rate was observed at 100 °C [142,144]. Some balanced reactions make the SEI undergo cyclical degradation and regeneration above 120 °C but below 250 °C [145]. The key reaction leading to this is shown below.

(1) $${\left(CH_{2}OCO_{2}Li\right)}_{2}\to Li_{2}CO_{3}+C_{2}H_{4}+CO_{2}+\frac{1}{2}O_{2}$$

This decomposition of the electrolyte is observed both in contact with graphite anodes and in the case of Li_4_Ti_5_O_12_ anodes [146,147,148]. The amount of graphite diminishes rapidly above the temperature of 205 °C, rendering a stoppage of balanced SEI decomposition and regeneration. This leads to reactions of the lithiated carbon with fluorine-based binders and of the solvent reaction with Li, once the temperature increases beyond 260 °C [142,144,149].

(2) $$-\left[CH_{2}-CF_{2}\right]-+Li\to LiF\pm \left[CH=CF\right]\mp \frac{1}{2}H_{2}$$

(3) $$2Li+RF_{2}\to 2LiF+\frac{1}{2}R_{2}$$

The reaction between solvent and any metallic lithium present (see below) is already observed at around 100 °C or even at a lower temperature of 68 °C for some special electrolytes [142,144].

(4) $$2Li+C_{3}H_{4}O_{3}\left(EC\right)\to Li_{2}CO_{3}+C_{2}H_{4}$$

(5) $$2Li+C_{4}H_{6}O_{3}\left(PC\right)\to Li_{2}CO_{3}+C_{3}H_{6}$$

(6) $$2Li+C_{3}H_{6}O_{3}\left(DMC\right)\to Li_{2}CO_{3}+C_{2}H_{6}$$

##### Cathode

The transition metal oxides acting as cathode material undergo breakdown, releasing oxygen at a temperature of around 200 °C in the case of LiCoO_2_ [150,151,152,153,154].

(7) $$Li_{y}CoO_{2}\to yLiCoO_{2}+\frac{1}{3}\left(1-y\right)Co_{3}O_{4}+\frac{1}{3}\left(1-y\right)O_{2}$$

(8) $$Co_{3}O_{4}\to 3CoO+\frac{1}{2}O_{2},CoO\to Co+\frac{1}{2}O_{2}$$

In the case of the Li[Ni_x_Co_y_Al_z_]O_2_ cathode material, the breakdown is already ob-served at around 140–160 °C [155,156,157,158].

(9) $$Li_{0.36}Ni_{0.8}Co_{0.15}Al_{0.05}O_{2}\to 0.18Li_{2}O+0.8NiO+0.05Co_{3}O_{4}+0.025Al_{2}O_{3}+0.372O_{2}$$

When LiNi_y_Co_x_Mn_z_O_2_ is used as cathode material, then the breakdown occurs only at around 260 °C and is followed by reactions that are exothermic in nature leading to a sharp increase in temperature [159,160,161,162].

(10) $$Li_{0.35}{\left(NiCoMn\right)}_{1/3}O_{2}\to Li_{0.35}{\left(NiCoMn\right)}_{1/3}O_{2-x}+\frac{x}{2}O_{2}$$

When using LiMn_2_O_4_ as the cathode material, the thermal breakdown is observed in-between 150 °C and 225 °C [163,164].

(11) $$Li_{0.2}Mn_{2}O_{4}\to 0.2LiMn_{2}O_{4}+0.8Mn_{2}O_{4}$$

(12) $$3Mn_{2}O_{4}\to 2Mn_{3}O_{4}+2O_{2}$$

(13) $$LiMn_{2}O_{4}\to LiMn_{2}O_{4-x}+\frac{x}{2}O_{2}$$

(14) $$LiMn_{2}O_{4}\to LiMnO_{2}+\frac{1}{3}Mn_{3}O_{4}+\frac{1}{3}O_{2}$$

(15) $$Mn_{2}O_{4}\to Mn_{3}O_{4}+\frac{1}{2}O_{2}$$

In the case of LiFePO_4_ as the cathode material, the breakdown is imminent between 190 °C and 310 °C [149,165,166].

(16) $$2Li_{x}FePO_{4}\to Fe_{2}P_{2}O_{7}+\frac{1}{2}O_{2}\left(x=0\right)$$

##### Electrolyte

Decomposition of the electrolyte is one of the most critical reasons for thermal runaway. The following reactions were observed from 200 °C to 300 °C [147].

(17) $$LiPF_{6}\to LiF+PF_{5}$$

(18) $$2ECLi-O-{\left(CH_{2}\right)}_{4}-O-Li+PF_{5}\to Li-O-{\left(CH_{2}\right)}_{4}+2LiF+POF_{3}$$

(19) $$C_{2}H_{5}OCOOC_{2}H_{5}+PF_{5}\to C_{2}H_{5}OCOOPF_{4}+HF+C_{2}H_{4}$$

(20) $$C_{2}H_{4}+HF\to C_{2}H_{5}F$$

(21) $$C_{2}H_{5}OCOOPF_{4}\to PF_{3}O+CO_{2}+C_{2}H_{4}+HF$$

(22) $$C_{2}H_{5}OCOOCPF_{4}\to PF_{3}O+CO_{2}+C_{2}H_{5}F$$

There is also another phenomenon that affects the thermal performance of the battery, which is the oxidation of electrolytes. This oxidation is initiated due to the breakdown of metal oxide cathode material releasing oxygen or due to the electrolyte coming in contact with air due to a damaged battery case [167].

(23) $$2.5O_{2}+C_{2}H_{4}O_{3}\left(EC\right)\to 3CO_{2}+2H_{2}O$$

(24) $$6O_{2}+C_{5}H_{10}O_{3}\left(DEC\right)\to 5CO_{2}+5H_{2}O$$

(25) $$3O_{2}+C_{3}H_{6}O_{3}\left(DMC\right)\to 3CO_{2}+3H_{2}O$$

(26) $$4O_{2}+C_{4}H_{6}O_{3}\left(PC\right)\to 4CO_{2}+3H_{2}O$$

Apart from these, even separators and collectors melt and thereby contribute to thermal runaway. In the case of separators, combinations of PP and PE are used, which have a melting temperature of 130–170 °C [84,89,168,169]. If they are coated with ceramic coatings, then their melting point may be pushed to a higher temperature range of 200–260 °C [164]. Collectors made of aluminum have a melting temperature of 660.3 °C (STP) and copper has around 1085 °C (STP) [8]. In summary, thermal management of batteries is very critical to keep the temperature within the operating range. Different methods are adopted to cool the battery packs, which are explained in detail in Section 5.3.

## 5. Mitigation Strategies

In this section, the possible mitigation strategies are discussed to overcome or restrict some specific modes and mechanisms of Lithium-ion battery failure. LiB safety is the prime focus, so multiple mitigation strategies are followed to keep the batteries safe. This can be done by two methods, one by avoiding operation conditions, which lead to heat and gas formation, the other being the process to control the heat and gas formation to avoid failure. More stable LiBs can be accomplished by alteration of the chemistry and/or component structures of the battery. The alteration can be made in electrodes and electrolytes [99]. The electrolyte alteration can be done by the addition of fire-inhibiting additives. The fire-inhibiting materials are non-flammable in nature and hinder the propagation of exothermic reactions [170]. The limitation of the magnitude of current flow during working cycles is also a mitigation strategy to keep the batteries safe. This restriction can be done by using POTC devices, which quickly respond to temperature changes.

**Figure 13:**
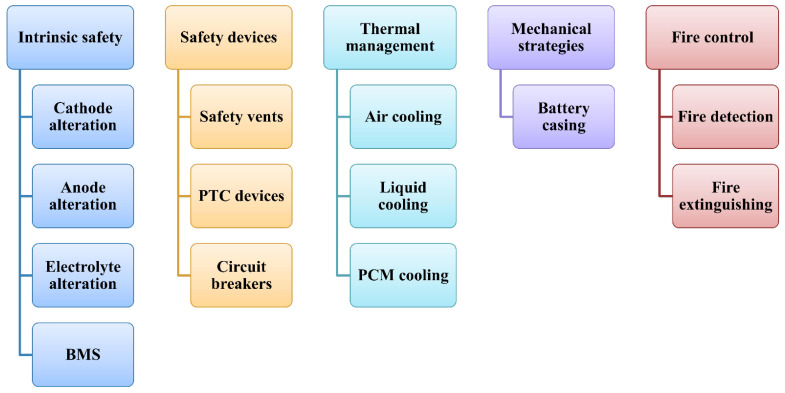
Classification of the main mitigation strategies implemented to achieve safety in Lithium-ion batteries.

The inclusion of dedicated safety devices to the battery design is also one of the mitigation strategies. These safety devices should be economical and should not lead to packaging issues in the battery [171]. The safety devices can be in the form of protection vents, current fuses, and additives in cell materials to shut the current flow. The electric fuses and contact assemblies shield the circuit from current fluctuation [172]. The mitigation of thermal runaway is particularly crucial, since postponing the thermal runway in the event of a battery failure gives the passenger more time to escape the harm which can be caused by the battery [167]. Thermal management of the battery is one of the most crucial mitigation strategies, as it will prevent hazardous heat accumulation in the battery pack. Thermal management can be achieved by air cooling, which is the simplest way of cooling, liquid cooling, which is most efficient but complex in design, and phase change cooling is best suited for extreme temperatures [173]. To suppress a fire after the failure of LiBs, various methods and extinguishing agents are used in electric vehicles. The most commonly used fire extinguishing agents are water, water mist, halons, etc., along with suitable additives to improve the properties of the extinguishers [99]. Overall, there are four main mitigation strategies with sub-classification as discussed above and shown in Figure 13. These mitigation strategies will be explained in detail below.

### 5.1. Innate Safety Strategies

#### 5.1.1. Anode Alteration (Protection)

**Figure 14:**
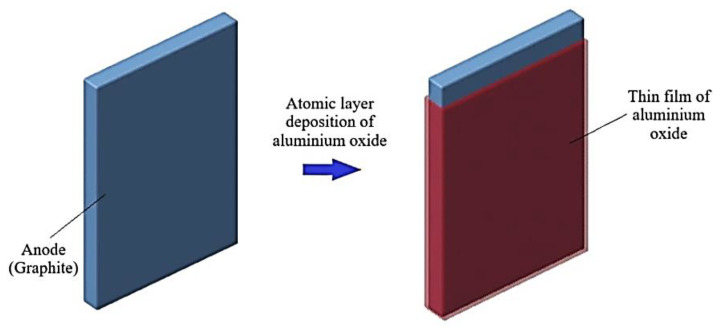
Atomic layer deposition process in the anode.

Surface coating is a popular method used for anode alteration. Among the coating technologies, atomic layer deposition (ALD) is widely used. A typical example is an Al_2_O_3_ coating carried out by the ALD process, which results in the formation of ultrathin conformal film covering the anode composite which improves both cycle performance and inertness towards thermal attack [174]. The process of ALD is as shown in Figure 14. The incorporation of thermo-responsive polymer microspheres on the material of the anode reduces the ionic conduction in the case of overheating by melting of the polymer layer when the temperature within the cell crosses critical value [175]. Likewise, the use of new thermally stable, higher voltage anode materials such as Lithium Titanium oxide (Li_4_Ti_5_O_12_) or Titanium niobates (TNO) instead of conventional carbon-based anodes improves the safety of the batteries [176]. Silicon nanowires are also used in anode electrodes which can withstand mechanical loadings [177].

Additives used in electrode composites enhance the temperature stability of the SEI layer built upon the anode surface. Higher temperature stability of the SEI layer, in turn, enhances the temperature activeness of the anode [178].

#### 5.1.2. Cathode Alteration

**Figure 15:**
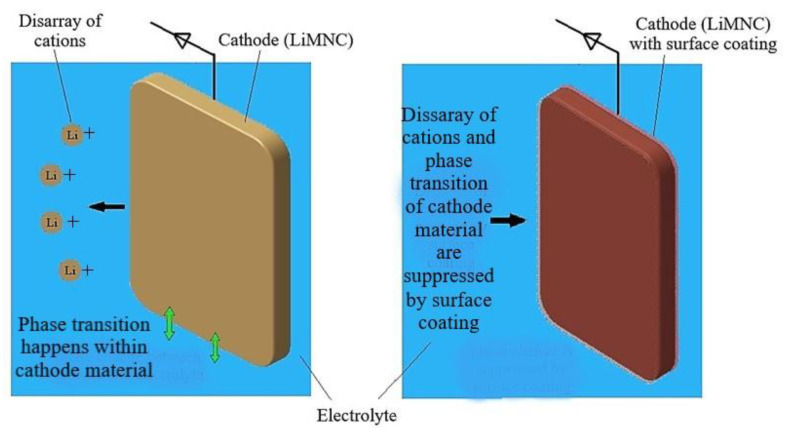
Surface coating in the cathode.

For cathode alteration, both surface coatings and element substitution are widely explored. The use of surface coating is one of the common methods to enhance the electrochemical performance of the cathode material. The coating materials have to be thermally and chemically inactive [166]. As per the study conducted by Li et al. [179], surface coatings obstruct the direct contact between the electrolyte and the cathode surface improves the structural stability, thereby suppressing phase transition of the active material, and restrain the disarray of cations. This is as shown in Figure 15. Iron phosphate (FePO_4_) coating improves the performance in electrochemical reactions and temperature stability of the cathode [180]. Tin oxide (TiO_2_) coating on a Li cathode enhances the cycling stability and discharge strength [181]. MnSiO_4_ is a common coating material on LCO that improves overcharge tolerance and increases thermal strength [99]. Fluorides are also preferred for their excellent electrochemical inertness at high temperatures. They can be employed in the form of AlF_3_, which improves cycling efficiency and reduces reactivity for side reactions under high-temperature stress [182]. A few other coatings, such as LiNbO_3_-coated NMC cathodes, are also in use.

**Figure 16:**
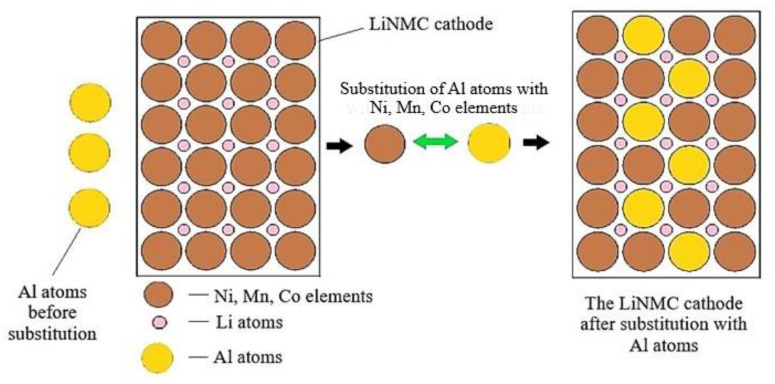
Element substitution process in the cathode.

The other important means of modification is element substitution, which is very effective in enhancing the performance of cathodes. Aluminum is one of the most efficient substitutions or doping agents for transition metals such as Co, Ni, and Mn, which increases the structural stability under thermal stress as sketched in Figure 16 at the expense of sacrificing some gravimetric capacity. A combination of Al and Ni can be used to increase the electrochemical performance [183].

#### 5.1.3. Electrolyte Alteration

**Figure 17:**
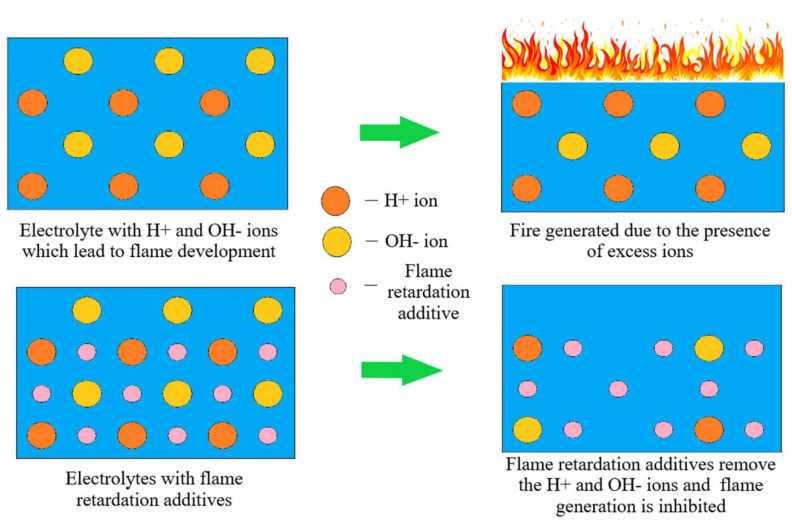
Removal of H^+^ and OH^−^ ions by adding flame retardation additives.

Additives added to the electrolyte enhance the intrinsic safety of lithium batteries by flame retardation, overcharge shielding, thermal inhibition, and SEI enhancement [167]. The flame retardation additives inhibit the fire-catching phenomenon during thermal runaway. Flame inhibitor additives are commonly made of phosphates (trimethyl phosphate, triphenyl phosphate, and dimethyl methyl phosphate) or halogenated compounds [184]. Their working principle is based on the removal of free radicals (e.g., H^+^ and OH^−^ ions) as indicated in Figure 17. The H^+^ and OH^−^ ions are formed due to the abstraction of hydrogen present in organic electrolytes. This process is driven by the highly reactive oxygen ions produced within the cell due to the high temperature caused during thermal runaway (the mechanisms are stated in [185]). Active ions are also formed by a chain branching reaction. Liquid flame retardation additives will release tiny radical species containing phosphorous or halogens during thermal runaway (the mechanisms are stated in [186]). These phosphorous or halogen radicals scavenge H^+^ and OH^−^ ions, thereby suppressing the exothermic chain reaction. Fire inhibition can also be achieved by the condensed state formation technique, which acts as an insulator to fire growth [187]. Although the flame-retardant additives suppress flame propagation by reducing the electrochemical performance of electrolytes, these additives must be used in calculated proportions. This is because a higher quantity of phosphates causes capacity fade and higher viscosity of alkyl phosphates reduces battery power and capacity utilization [188]. Zeng et al. developed dimethyl methyl phosphonate (DMMP) as an electrolyte solvent and concluded that this new solvent provides excellent electro-chemical compatibility with electrodes and improved nonflammability properties [189]. The non-halogenated additive methoxyethoxyethoxyphosphazene oligomer can increase the fire resistance of the electrolyte and also retain its high electrochemical performance [170]. Additives such as fluorinated cyclotriphosphazene and vinylene carbonate can also improve temperature inertness. The specific SEI layer formed under the influence of these additives protects the cell constituents from thermal damage [190]. Alternatively, Lithium phosphorous oxynitride is one promising solid electrolyte that, when employed as an artificial SEI, inhibits fires and can stop the dendrite formation [23].

#### 5.1.4. Separators

The most common separators are made of Polyethylene (PE) and Polypropylene (PP). A shutdown type of separator is made up of multilayer films which help in closing the pores and prevent the ion transport across the separator during excess thermal runaway. The combination of PE and PP layers improves the safety of the separator, as the PE layer will melt and fill the pores of the PP layer by the creation of a protective film that limits the flow of current during cell abuse [191]. This irreversible restriction of current flow explains the name ‘shutdown separators’. Even after a successful shutdown, the temperature will continue to rise (due to the inertia effect), which will often lead to a subsequent melting of the separator and a consequential short-circuiting of the electrodes. Therefore, there should be a considerable difference between the shutdown and meltdown temperature of the ‘shutdown separator’ to ensure its effectiveness. Extensive research is also made on tri-layer separators, in which the melting temperature of the central layer is lower than for the two outer layers. The greater time lag between reaching the shutdown temperature and reaching the temperature of shrinking or melting buys time for additional measures to control thermal runaway [192]. Ceramic coatings added to polyolefin separators will enhance melting temperature and wettability along with a significant amount of reduction in material shrinkage at high temperatures [193,194]. To prevent dendrite growth in the anode, the separator surface facing the anode is normally coated with an ultrathin film of copper. This serves as a conducting agent to prevent electrically isolated Lithium on the anode surface. Furthermore, the electrically conductive nature of Cu guides the rear side plating of Li metal and modulates its deposition morphology through the merging of dendrites [195]. Terephthalate-based separators are alternatives to the commonly used polyolefin-based materials. These separators are made of polybutylene terephthalate (PBT) which enhances the thermal stability of the entire cell by closing the gap between the separator phase transition and cell runaway temperature. The key properties of this type of separator are better permeability and electrolyte wettability with commonly used carbonate-based electrolytes. These PBT-based separators are good at withstanding cell abuse [90].

#### 5.1.5. Battery Management Systems

Despite LiBs being the most viable option to power electric vehicles, they have issues that need to be tackled to ensure optimum performance. The battery works at peak efficiency only when maintained at a certain temperature range (20–40 °C (±5 °C)). Previously discussed issues such as ISCs and thermal runaways can be monitored as well as prevented to a certain extent by using Battery Management Systems (BMS). In the case of monitoring the battery’s health, the BMS plays a vital role. It monitors parameters and stats such as State of Charge (SOC), State of Health (SOH), Depth of Discharge (DOD), and State of Function (SOF) for the entire pack as well as for individual cells.

BMS components can be divided into two major categories: software and hardware [196]. The software component includes data collection and estimation, onboard diagnostics, charge control, and cell balancing. The hardware part comprises multiple sensors to track battery parameters, safety circuits to avoid battery over-charging, over-heating and over-discharging, galvanostat, and potentiostat for cell balancing. Data logging is done at fixed intervals to constantly monitor the battery’s health [197]. Parameters that are monitored are the voltage of cell and pack, temperature and requirement of isolation and interlocks [198]. If any abnormal parameter values occur, the BMS will alert the user or operator regarding the same using inbuilt communication protocols and devices. The BMS is also responsible for protecting the battery pack from potential hazards by anticipating them using the data collected and guiding the system back to safe territory. These events, uncontrolled, eventually could lead to battery failure and may be either intrinsic or extrinsic in nature. BMS are the decision-makers regarding the use of relevant avant-garde methods and mechanisms that help in safeguarding the battery from probable perilous scenarios and operating conditions [198].

The role of BMS for an easier understanding can be further classified as system management, identification, or prediction of an endangering scenario, and the response and decisions to be taken once the system’s abnormal behavior is observed [199]. Firstly, in the case of system management, BMS’s role is to monitor the various operations, detecting and setting up a Safe Operating Area (SOA) for each cell, deciding the fault criteria, and finally identification and authentication of system. In the next type, i.e., while detecting or predicting a perilous scenario, the parameters affecting the safety of the system are further classified depending on whether the affecting parameter is either intrinsic or extrinsic to the pack. In the intrinsic case, the electrical parameters of a cell are mostly taken into consideration and in the case of the external case, the key parameters to be monitored are overall pack level parameters. Apart from its decision-making towards predicting failures, BMS should run fault diagnostic checks regularly, along with the managing of thermal effects, the managing working cycles, and also update the main vehicle control system regarding the onboard events occurring in the system [199]. Once a fault is identified, it is required to take necessary countermeasures. These countermeasures involve isolating the faulty cell/cells or even an entire module depending on the severity of the damage. Furthermore, the user is informed of the damage and a limp home mode is engaged to minimize or avoid further damage. If the severity of the fault requires, the entire battery pack is cut off from the electrical system.

### 5.2. Protective Devices

#### 5.2.1. Protection Vents

**Figure 18:**
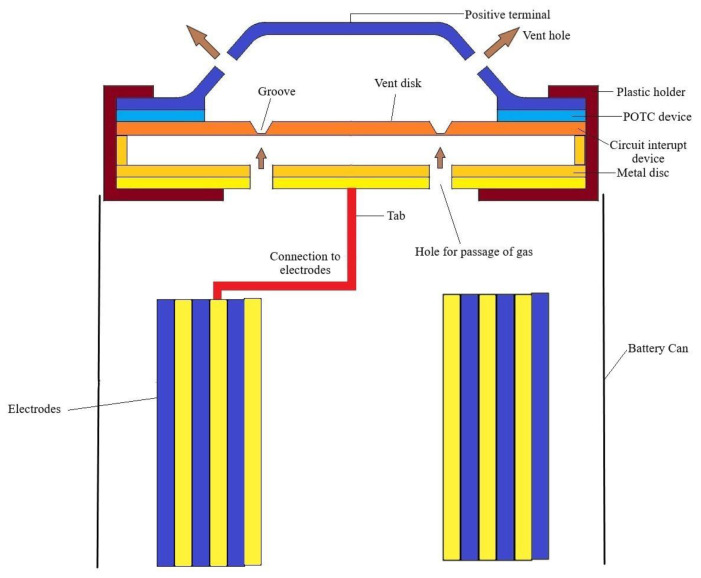
The vent holes and POTC device in the assembly of a Li-ion cell. Reprinted from refs. [201,202].

Protection vents act as a response mechanism to release excess pressure developed inside the battery beyond the threshold by letting the gas escape into the atmosphere. The escaping gas is typically mainly CO_2_ and solvents, but will also contain HF, POF_3_ and in the case of a fire, the combustion products are formed from above. A mechanism can be designed with a mechanical link using a metal disc, which is attached to the cathode. This breaks the circuit and makes way for the gases to escape. The protection vent can be seen in Figure 18, in the Li-ion cell assembly. To test this mechanical link, Kato et al. [200] deliberately added lithium carbonate to the cathode and charged the system to a voltage greater than 4.8 V. The added lithium carbonate reacts with the deintercalated Li_x_CoO_2_ at higher voltage releases of carbon dioxide within the system and hence the pressure was built up. This actuated the aluminum disc, which disconnected the cathode from the circuit there by protecting the internal components. This protection vent acts as a backup safety device as it is irreversible and accompanies the release of organic compounds which may pose additional risks that need to be managed. Usually, the POTC will overrun the operation of the protection vent by reversibly limiting the current in the circuit under overheating conditions, as described below [171].

#### 5.2.2. Positive Thermal Coefficient Device (POTC)

POTCs typically consist of conductive polymers and operate on the principle that heating caused by the joule effect correspondingly increases the resistance in the device. This will strongly, but reversibly, inhibit the current passage if the cell is overheated. Under normal operation temperatures, the POTC should contribute only negligibly to the overall cell resistance [172]. The POTC device is shown in Figure 18. The standard 18,650 cells have POTC devices whose surfaces are metal-coated, which enclose PE layers mixed with carbon black [203]. The POTC can be classified as a ceramic and polymer type. The ceramic type POTC is suitable to work at a high voltage and is suited for high-voltage circuits. The conductive polymer-type POTCs are non-linear POTCs, which are made of composite polymers, such as e.g., PE coated with acetylene black [164,165].

#### 5.2.3. Other Circuit Cut off Devices

Some of the other (typically reversible) circuit cut-off devices include magnetic switches, bimetallic thermostats, and electronic Protection Circuit Modules (PRCM), which can be used to shield the battery pack, preventing the need to trigger irreversible shutdown mechanisms. The PRCM is attached to the battery circuit independently. When there is a malfunction in the circuit, the PRCM will break and the circuit is shielded [204]. Thus, LiBs need an overcharging protection circuit. This can be done by a control unit. Thermal fuses are the oldest and still most common current inhibitor device. It is a one-shot fuse that consists of a wire, which is made of a fusible metal or alloy. The principle is based on the melting when the current reaches the threshold level and sufficient heat is generated to melt. The advantage of this device is its simple construction; it is economical and easily designed for a wide range of voltage and current levels. The fuse wire is connected in series to the battery pack and the melting point is above the maximum operation temperature [171].

### 5.3. Thermal Management

As discussed in Section 4.3, LiBs operate efficiently in certain temperature ranges and hence thermal management of batteries plays a key role in maintaining battery health. The strategies involved in a battery thermal management system (BTMS) can be classified based on many factors, such as the control methodologies applied, their position, or the type of contact to the heat source. Based on control methodologies, there are two types, namely active and passive cooling; in terms of position, there are internal and external cooling and by contact, it is indirect and direct. According to the cooling medium, the BTMS may be classified as air cooling, liquid cooling, phase change material cooling, and heat pipe cooling.

#### 5.3.1. Air Cooling of LiBs

Air cooling is the most popular method for small battery packs where the heat generated is not so high. There are different methods of air cooling of LiBs in terms of contact, namely direct air cooling and indirect air fin cooling. Based on the type of energizing air to pass through, there is forced air and free air-cooling. A pump is utilized to force the air from the atmosphere to the battery packs in the forced mode of air-cooling. The forced mode of air cooling is mainly used when a large amount of heat is to be removed from the battery packs, or when there is no duct present in the vehicle for air passage from the atmosphere. In free air cooling, the vehicle motion drives the passage of air into the battery packs through proper ventilation [205].

In the direct mode of air-cooling, the air is passed directly over the surface of the cells through the tubes or ducts. As the air passes the exposed surfaces of the battery, the heat is taken away by the airflow via convective heat transfer. The effectiveness of the cooling system is based on several parameters, such as the heat transfer coefficient of air and the battery surface, which exposed the face area of the batteries in contact with airflow [205]. In indirect air cooling, a metallic heat sink is used as the fin material, which provides a larger surface area, and as they are exposed to the atmosphere, the heat from the battery cells is transferred to the fins by conduction and surrounding air takes away the heat from the exposed fin surfaces through convection [206]. This method of air cooling has the advantages of a low cost, lighter packaging, and a simple design [197], but is best suited for small battery packs, e.g., in two-wheelers, where the amount of heat to be removed is limited.

#### 5.3.2. Liquid Cooling of LiBs

Liquid cooling is always considered a better alternative to air cooling, especially for larger battery packs, because of their higher thermal conductivity and the high specific heat of the coolant compared to the air [207]. Liquid cooling can be classified as internal and external cooling.

Internal cooling is the process [207] where the cooling system is implemented inside the battery pack. This cooling method is very efficient, and the heat is transferred to the coolant from its origin by a microchannel evaporator, which is sandwiched between two separate collectors. The advantage of this method is that buoyancy drives the circulation of the coolant liquid. As an alternative (e.g., for data centers or transformer stations), immersion cooling is explored, where a sufficiently stable dielectric coolant is circulated throughout the energy storage module in contact with cell walls, tabs, and electrical wiring.

External cooling refers to cooling by a liquid that is passed along the outer periphery of the battery pack. Among its advantages are low cost and flexibility in design. Different equipment used here can be in the form of tubes, jackets, and channels designed according to the battery layout [207,208]. The supplementary components which carry the coolant create a negative impact on heat conduction. External cooling is considered a safer alternative, as it evades direct contact between coolant and the battery surface [207].

The selection of an appropriate coolant is very critical to obtain better efficiency. Major coolants include water, oil, nano-fluids, and liquid metals. Water is mainly utilized in various cases of cooling. It possesses better properties in terms of specific heat capacity, thermal conductivity, and viscosity. Water consumes less power and is circulated through pads, jackets, and tubes to avoid the short circuit that might occur if it comes in contact with the battery terminals [209]. Oil is another alternative, which is mostly used for an immersed type of cooling system. The efficiency, in this case, is the highest and the packaging is easier. Due to higher viscosity, more pumping power is required to push the fluid to flow but can give three times better cooling than its counterpart because of direct contact of the fluid with the battery [173]. Nano additives will enhance the properties of water or glycol in terms of thermal conductivity. Common nano additives are Al_2_O_3_, CuO, Fe_3_O_4_, TiO_2_, Al, Cu, Ni, Ag, and other metals or metal oxides. The thermal conductivity of metals is higher compared to standard cooling fluids. The addition of these particles into the cooling fluid can increase its thermal conductivity to a large extent. The impact of an additive on the thermal conductivity is related to the atomic size. In general, the lower the atomic radius, the higher the thermal conductivity [207].

#### 5.3.3. Phase Change Material (PCM) Cooling of LiBs

PCM cooling is another alternative that is used for LiBs cooling. The PCM materials possess a high latent heat of fusion and ideal fusion point. The flow pathway of heat in the battery of PCM cooling is that the heat generated in the battery is passed to PCM and it reduces the maximum of the heat without passing it to further materials such as the battery casing. The concept of PCM is that when the heat is absorbed by the material there is a phase change from solid to liquid and as soon as that heat is pulled out of the system the PCM material changes its state back to solid [210].

The key property of PCM material is a suitable melting temperature within the range of operation of the battery. This is very critical to achieve effective heat dissipation. The packaging of the battery pack has to be done carefully with additional volume available for the PCM material when it changes to the liquid state. To tailor the heat transfer properties of PCM materials, a metal matrix can be embedded, or high thermal conductivity additives and additional fins for heat removal can be added. The system can also be designed with a dual function as a latent heat storage system. The PCM approach has an advantage in terms of weight and flexibility in the packaging of the battery pack [211]. Common PCM materials are paraffin, fatty acids for low-temperature applications, and molten salts for high-temperature applications. Examples of low-melting metal salts used in this application are sodium sulfate, potassium hydroxide, sodium chloride with potassium chloride, etc. In automotive applications, some of the most widely used PCM materials, especially for a hybrid electric vehicle or electric vehicle application, are PCM with aluminum foam, graphite matrix, or copper foam to optimize heat conduction [210].

### 5.4. Mechanical Mitigation Strategies

**Figure 19:**
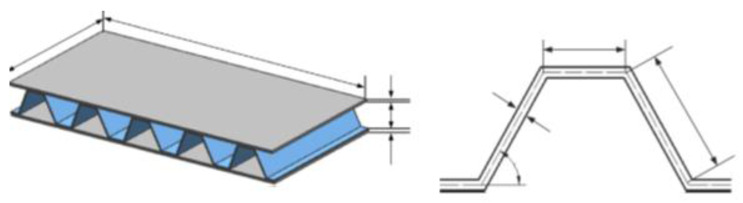
Cross-sections of NavTruss sandwich structure. Reproduced with permission from ref. [215]. Copyright 2013. Elsevier Ltd.

The most basic mitigation strategies for different mechanical failures due to compression and penetration of the batteries are to optimize the battery casings. These casings can adopt different cross-sections like the double layer metal sheet, NavTruss (as shown in Figure 19), BRAS, etc. [212] The battery casings shield the battery from mechanical forces by the principle of absorption of the kinetic energy by compression of the structure. The main parameters which influence the energy absorption are the stroke length, peak force, and energy absorption [213]. Zhu et al. [212] conducted a simulation where a comparison of the shortening length of different structures, namely double-layered, NavTruss, and BRAS, was done. In comparison, BRAS yielded the lowest shortening length, which implies that it has the maximum kinetic absorption capability when compared to other structures [212]. Similarly, Halimah et al. found from nonlinear finite element methods simulations comparing NavTruss and BRAS structures [214] that the energy absorbed by the BRAS structure is higher. The structural phenomenon involved in the structures is progressive buckling in BRAS and small bending in NavTruss [213]. The structures like BRAS and NavTruss can be used for shielding the battery packs against impact loads from the bottom or side of the vehicle.

### 5.5. Fire Control

#### 5.5.1. Fire Diagnosis Device

Fire propagation has become more prominent and can cause devastating damage. Plenty of research is focused on the early diagnosis of fire in the battery pack and the addition of fire suppressors to limit the damage caused. Common diagnosis devices are heat detectors, smoke detectors, and a combination of both which diagnose the ignition in the LiBs. Research finds that smoke detectors are more apt for this solution when compared to fire detectors as the response time of smoke detectors is much shorter [99].

#### 5.5.2. Fire Extinguishing

Fires generated during the failure will not depend on external oxygen to burn actively, but rather are fed by internal reactions within the cell. Water is one of the cheapest and abundantly available agents to suppress fire [99]. During this suppression, fluorides from the electrolyte of the cell will react with water and can produce HF, which is toxic to human health [216]. Lithium can also reduce the water to flammable hydrogen gas. Water mist has advantageous inhibition characteristics and cooling efficiency, but the spray time of the water mist has to be more and should almost be equivalent to the time elapsed from the initial rupture of the cell. The addition of a surfactant can aid the water mist in absorbing flammable gasses, such as methane and carbon monoxide [99]. Compared with water or water mist, their combination with fire-fighting foams and powders has a higher fire repression efficiency [217].

**Figure 20:**
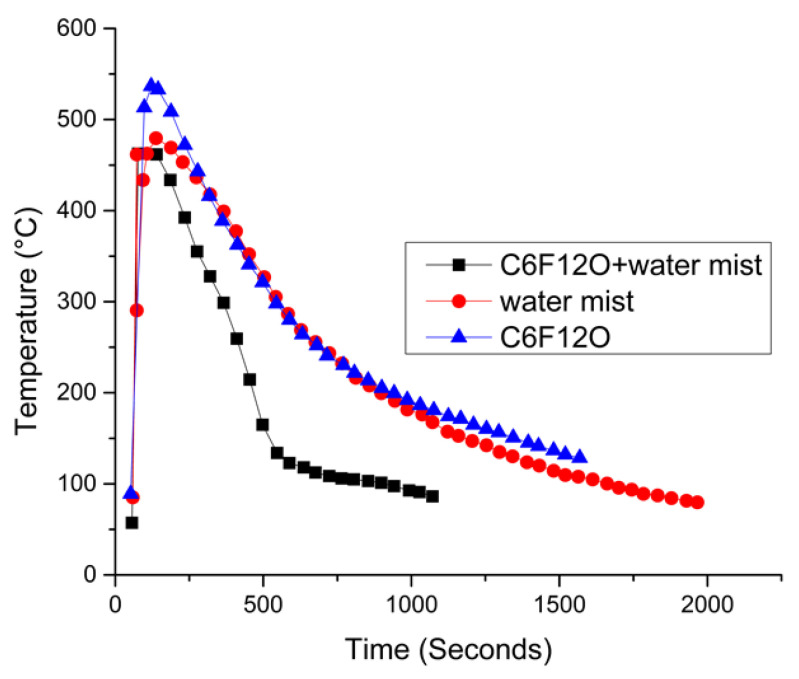
Temperature response of Li-ion battery surface upon application. Reproduced with permission from ref. [218]. Copyright 2019. Elsevier Ltd.

The gaseous suppression agent dodecafluoro-2-methylpentan-3-one (C_6_F_12_O) is a commercially used agent to enhance fire suppression. Figure 20 compares the maximum temperature reached and the time taken to reduce the temperature with water mist, C_6_F_12_O, and a combination of both. It can be observed that the combination of C_6_F_12_O with the water mist will reduce the peak temperature of the surface and also reduce the temperature in the shortest interval of time [218]. ABC powder, carbon dioxide, and aqueous solution were able to curb open flames in the LiBs [99]. Halons are also used to repress fire, but the drawback is that as soon as the repression stops, the temperature of the cell increased again. Therefore, the halons did not prevent the re-ignition of fire. The usage of halons is reduced as it is harmful to the ozone layer of the earth [219]. Heptafluoropropane is another extinguishing agent which can be used to fight fires in small battery packs. The repressed fire might reignite due to the exothermal chemical reactions that take place inside the battery when the agent is applied. Once again, the application of this agent has to be in the early stages of fire development [220]. Though several safety systems are implemented in electric vehicles, fire accidents still exist and they cannot be avoided completely due to unpredictable field failures and the high energy density desired.

## 6. Summary

This review summarizes materials, failure modes and mechanisms, and different mitigation strategies that can be adopted for the improvement of Lithium-ion battery safety. NMC and LFP are promising cathode materials. Moving forward, graphite is commercially widely used as an anode material. However, research is going on to implement lithium metal or silicon as an anode owing to its advantages. In the area of electrolytes, the focus is on solid and gel polymer electrolytes. Furthermore, different mechanisms and modes of failure were studied using the FMMEA method. It was observed that anode active material is more susceptible and plays a critical role in leading to system-wide failure. Therefore, this component needs to be re-worked and its safety should be improved. In the case of modes, mechanical is one of the most perilous, as it rapidly deteriorates the health of the battery, simultaneously leading to other modes of failure. Understanding failures will help us to formulate mitigation strategies. Among them, innate safety strategies include alterations made in cell materials where additives are coated on the electrodes which will aid the cell performance; additives are also added to electrolytes, which make the electrolytes fire-resistant. Protection devices are introduced to shield the battery pack from variation current passage. In the thermal management of battery packs, different strategies are used in different applications, such as air cooling used in small battery packs with less heat generation, liquid cooling used in large battery packs with higher heat generation and PCM cooling can be used in small battery packs with packaging constraints. All these strategies are used to evade the excessive heat generated in the batteries. The fire control strategy is used to suppress the fire after the lithium-ion battery fails. It includes fire diagnosis devices like smoke detectors, which have a better response time compared to fire detectors, and in extinguishing agents, water mist with suitable additives is a viable option as it is available in abundance and has fewer side effects during the process of extinguishing.

## Figures and Tables

**Table 1:** Modes of contact and its effects (based on the data from [140]).

Contact Mode	Resistance to ISCs	Temperature Rise	Heat Generation	Heat Conductivity	Severity of Failure	Probability of Occurrence
Anode-Current collector (Cu-Foil)	Low	Low	High	High	Most dangerous	No data available
Cathode-Anode	High	High	Comparatively low	Low	Second most dangerous	Maximum
Current collector- Cathode (Al–foil)	High	Low	Least	High	Low	Minimum to rare
Current collector- Current Collector	Low	Low	Highest	Highest	No data available	Low

## Data Availability

Not applicable.

## Abbreviations

ALD	Atomic Layer Deposition
ARC	Accelerated Rate Calorimetry
BMS	Battery Management System
BRAS	Blast Resistant Adaptive Sandwich
BTMS	Battery Thermal Management System
DEC	Diethyl Carbonate
DMC	Dimethyl Carbonate
DOD	Depth of Discharge
DSC	Differential Scanning Calorimetry
EC	Ethylene Carbonate
EMC	Ethylmethyl Carbonate
FIB	Focused Ion Beam
FMEA	Failure Mode Effects Analysis
FMMEA	Failure Mode Methods Effects Analysis
FTIR	Fourier Transform Infrared Spectroscopy
ISCs	Internal Short Circuits
LCO	Lithium Cobalt Oxide
LFP	Lithium Iron Phosphate
LiBs	Lithium-ion batteries
LMO	Lithium Manganese Oxide
LTO	Lithium Titanate
NavTruss	Navy Truss
NCA	Lithium Nickel Cobalt Aluminium
NMC	Lithium Nickel Manganese Cobalt
NMR	Nuclear Magnetic Resonance
PAN	Polyacrylonitrile
PBT	Polybutylene Terephthalate
PC	Propylene Carbonate
PE	Polyethylene
PET	Polyethylene Terephthalate
PoE	Polymer Electrolyte
POF	Physics of Failure
POTC	Positive Thermal Coefficient Device
PRCM	Protection Circuit Modules
PP	Polypropylene
PCM	Phase Change Material
PVdF	Polyvinylidene Fluoride
SEI	Solid Electrolyte Interface
SEM	Scanning Electron Microscope
SOA	Safe Operating Area
SOC	State of Charge
SOF	State of Function
SOH	State of Health
TGA	Thermo-Gravimetric Analysis
ZS	Zero Strain
